# Transcription factors GATA-4 and GATA-6 in normal and neoplastic human gastrointestinal mucosa

**DOI:** 10.1186/1471-230X-8-9

**Published:** 2008-04-11

**Authors:** Hanna Haveri, Mia Westerholm-Ormio, Katri Lindfors, Markku Mäki, Erkki Savilahti, Leif C Andersson, Markku Heikinheimo

**Affiliations:** 1 Children's Hospital, University of Helsinki, Helsinki, Finland; 2 Institute of Biomedicine, Biomedicum Helsinki, University of Helsinki, Helsinki, Finland; 3 Pediatric Research Center, Medical School, University of Tampere, Tampere, Finland; 4 Department of Pediatrics, Tampere University Hospital, Tampere, Finland; 5 Department of Pathology, Haartman Institute, University of Helsinki, and HUSLAB, Helsinki University Hospital, Helsinki, Finland

## Abstract

**Background:**

Human gastrointestinal mucosa regenerates vigorously throughout life, but the factors controlling cell fate in mature mucosa are poorly understood. GATA transcription factors direct cell proliferation and differentiation in many organs, and are implicated in tumorigenesis. GATA-4 and GATA-6 are considered crucial for the formation of murine gastrointestinal mucosa, but their role in human gastrointestinal tract remains unexplored. We studied in detail the expression patterns of these two GATA factors and a GATA-6 down-stream target, Indian hedgehog (Ihh), in normal human gastrointestinal mucosa. Since these factors are considered important for proliferation and differentiation, we also explored the possible alterations in their expression in gastrointestinal neoplasias. The expression of the carcinogenesis-related protein Indian hedgehog was also investigated in comparison to GATA factors.

**Methods:**

Samples of normal and neoplastic gastrointestinal tract from children and adults were subjected to RNA *in situ *hybridization with ^33^P labelled probes and immunohistochemistry, using an avidin-biotin immunoperoxidase system. The pathological tissues examined included samples of chronic and atrophic gastritis as well as adenomas and adenocarcinomas of the colon and rectum.

**Results:**

GATA-4 was abundant in the differentiated epithelial cells of the proximal parts of the gastrointestinal tract but was absent from the distal parts. In contrast, GATA-6 was expressed throughout the gastrointestinal epithelium, and in the distal gut its expression was most intense at the bottom of the crypts, i.e. cells with proliferative capacity. Both factors were also present in Barrett's esophagus and metaplasia of the stomach. GATA-6 expression was reduced in colon carcinoma. Ihh expression overlapped with that of GATA-6 especially in benign gastrointestinal neoplasias.

**Conclusion:**

The results suggest differential but overlapping functions for GATA-4 and GATA-6 in the normal gastrointestinal mucosa. Furthermore, GATA-4, GATA-6 and Ihh expression is altered in premalignant dysplastic lesions and reduced in overt cancer.

## Background

The structure of human gastrointestinal mucosa is highly complex. The mucosal morphology and enzymatic function are reached mainly during mid-gestation, but the mucosa undergoes regeneration throughout life. The mature mucosa comprises multipotent stem cells, their immediate progeny, and various differentiated cell types that serve digestive, absorptive, or immunologic functions. The maintenance of mucosal homeostasis requires a balance between rapid regeneration and cell turnover. Little is known, however, about the factors determining tissue renewal and cell fate in the gastrointestinal tract.

GATA proteins are zinc finger transcription factors that regulate cellular development and differentiation. The GATA family contains six members; GATA-1, GATA-2, and GATA-3 function mainly in hematopoietic cell lineages [1], whereas GATA-4, GATA-5, and GATA-6 are found in organs of endodermal origin [2]. In the gastrointestinal tract, GATA-4 and GATA-6 are present from the onset of formation of the primitive gut tube through the organization of normal postnatal epithelium [3-6]. GATA-4 is considered to be one of the first transcription factors binding to chromatin during early endodermal differentiation [7]. This binding has been proposed to initiate the opening of the chromatin and enable the binding of other transcription factors to DNA [8]. Mice with targeted deletion of *Gata4 *or *Gata6 *die during early embryonic development: GATA-6 is crucial in the development of visceral endoderm, whereas GATA-4 is needed for the formation of the heart tube [4,9].

GATA-4 and GATA-6 are both believed to facilitate the terminal differentiation of the epithelium of the intestinal villi [10]. In mice chimeric for *Gata4 *-/- and wild type cells, the gastric epithelial lineages were found to differentiate poorly during embryonic development [6]. In mice harbouring a tissue-specific deletion of *Gata4 *in the gut, jejunal gene expression was noted to change to the ileal-type, underscoring the role of GATA-4 in the development of the proximal small intestine [11]. In the gastrointestinal tract, GATA proteins regulate a variety of target genes involved in digestive functions and tissue regeneration. For example, two genes expressed in enterocytes of the small intestine, lactase phlorizin hydrolase (LPH) and fatty acid binding protein (FABP), are regulated by GATA-4, GATA-5, and GATA-6 [12-15]. Moreover, GATA factors are related to gastroprotective Trefoil Factor Family (TFF) peptides and mucin protein genes expressed in the stomach and colon mucosa [16,17].

In addition to normal growth, GATA proteins have been proposed to be associated with tumorigenesis in the adrenals, lungs and ovaries [18-20]. GATA-6 upregulates expression of Indian hedgehog (Ihh) [21]; the hedgehog signalling is known to be critical for proper gut morphogenesis [22]. Ihh is present in mature human gastric and colon epithelium [23,24], and its expression is altered during carcinogenesis of the gastrointestinal tract [23-25]. The role of Ihh in tumor biology, however, remains controversial. Ihh is believed to stimulate proliferation and induce carcinogenesis in the gastrointestinal tract [25,26], but it may also act as an antagonist of the Wnt-pathway to restrict tumor progression [23].

In this study we compare the expression of GATA-4, GATA-6, and Ihh in normal and pathological human gastrointestinal tissue samples. We show that GATA-4, GATA-6 and Ihh have particular expression patterns in the gastrointestinal mucosa, depending on the segment in the longitudinal or crypt-villus axis, as well as the specific cell type. In addition, the expression patterns of these factors in gastrointestinal neoplasms differ from those in the normal gastrointestinal tract. We suggest that GATA-4 and GATA-6 may maintain normal tissue renewal and differentiation in mature gastrointestinal mucosa and that alterations in their expression accompany premalignant and malignant changes in the gut mucosa.

## Methods

### Normal samples

Normal gastrointestinal biopsy specimens were obtained during esophagogastroduodenoscopy and colonoscopy performed for diagnostic purposes. The specimens (Table 1) included tissue from gastric, duodenal, ileal, colonic and rectal mucosa. Most of the samples (37 out of 55) were collected from children. All biopsy specimens were diagnosed as morphologically normal by a pathologist.

**Table 1:** Patients' ages and the number of normal gastrointestinal samples as to the methods used

Tissue	Age (yr) Range (median)	GATA-4		GATA-6		Ihh^3^
		IHC^1^	ISH^2^	IHC^1^	ISH^2^	IHC^1^
Stomach	0.95 – 91.0 (15.4)	11	6	10	7	7
Duodenum	0.95 – 73.0 (11.6)	16	9	14	8	8
Ileum	6.08 – 76.0 (31.0)	8	3	7	3	4
Colon	5.90 – 76.0 (13.9)	8	6	7	6	2
Rectum	0.04 – 13.91 (6.89)	6	9	3	8	1

Abbreviations: ^1^IHC = immunohistochemistry, ^2^ISH = in situ hybridization, ^3^Ihh = Indian hedgehog

### Pathological samples

The pathological samples from distal esophagus and stomach were collected during routine esophagogastroduodenoscopy from adult patients with chronic and atrophic gastritis (Table 2). Esophageal biopsies contained intestinal metaplasia (also called Barrett's esophagus), a precancerous state of the esophageal epithelium. Neuroendocrine cell hyperplasia was found in three stomach samples. Colon and rectum samples were obtained in resections for adenomas and primary adenocarcinomas. Some of them contained dysplasia (n = 5) and invasive carcinoma (n = 2).

**Table 2:** Description of the neoplastic samples subjected to GATA-4, GATA-6 and Ihh immunohistochemistry

Tissue	Diagnosis	Age (yr) median (range)	Number of samples
Esophagus			
	Barrett's esophagus	65 (55–83)	9
Stomach			
	Chronic gastritis, intestinal metaplasia	67 (56–73)	5
	Chronic gastritis, intestinal metaplasia, neuroendocrine cell hyperplasia	52 (49–54)	2
	Intestinal metaplasia, neuroendocrine cell hyperplasia	69	1
Colon			
	Dysplasia	41	1
	Adenoma serratum, dysplasia	77	1
	Adenoma tubulovillosum, dysplasia	47 (28–73)	3
	Adenocarcinoma, low grade	54 (41–87)	3
	Adenocarcinoma, moderate grade	71 (47–79)	5
	Adenocarcinoma, high grade	76 (72–80)	2
Rectum			
	Adenocarcinoma, high grade	83	1
	Adenocarcinoma, moderate grade	79	1

### Tissue preparation and ethical considerations

Tissue specimens, originally collected for diagnostic purposes, were fixed in formalin and embedded in paraffin for further investigations, then cut into 6-μm sections, coded, and evaluated; the evaluator had no knowledge of the specimen. The specimens used for *in situ *hybdization experiments were frozen in liquid nitrogen and stored at -70°C until used. The use of the samples in this study was approved by the Ethics Committee of the Hospital for Children and Adolescents and the Ethics Committee of the Department of Internal Medicine, Helsinki University Central Hospital, and the Finnish National Authority of Medicolegal Affairs.

### In situ hybridization

Radioactive *in situ *hybridization was performed on normal gastrointestinal samples (Table 1). Paraffin embedded tissue sections (n = 22) were deparaffinized, and frozen sections (n = 9) were fixed in 40 g/L paraformaldehyde in phosphate-buffered saline (PBS). We applied the procedure described earlier [27]. Human GATA-4 and GATA-6 cDNAs were prepared as previously described [28]. Tissue sections were incubated with antisense or sense riboprobes labeled with ^33^P (1.2 × 10^6 ^cpm, 1000–3000 Ci/mmol, Amersham Pharmacia Biotech, Arlington Heights, IL) in a total volume of 80 μl. The slides were assessed by three researchers independently and blinded under a dark field light microscope (Leica DMRXA microscope, Leica, Switzerland).

### Immunohistochemistry

Deparaffinized sections of normal and pathological tissue (Tables 1 and 2) were dehydrated, and antigen retrieval was improved by microwave cooking for 20 min in 10 mM citric acid, pH 6. The endogenous peroxidase reaction was inhibited by treatment with 3 g/L of H_2_O_2_. The sections were then subjected to immunohistochemistry, using polyclonal goat anti-human GATA-4 (sc-1237, dilution 1:200) or Ihh (sc-1196, dilution 1:50) antibodies, or polyclonal rabbit anti-human GATA-6 antibody (sc-9055, dilution 1:50). The antibodies were from Santa Cruz Biotechnology (Santa Cruz, CA). The specificity of the staining was assessed using preimmune serum or PBS instead of the primary antibody during the staining protocol. In addition, our earlier studies with consecutive samples for RNA in situ hybridization and immunohistochemistry on adrenal samples have revealed the reliability of the used anti-GATA-4 and anti-GATA-6 antibodies in human tissues [28]. In some instances, GATA-6 gives a cytoplasmic staining as previously reported [15]. Although cytoplasmic staining for GATA proteins usually has not been considered specific, there is also some evidence that GATA proteins are not always transported to the nucleus immediately after translation [29]. The avidin-biotin immunoperoxidase system (Vectastain Elite ABC Kit, Vector laboratories, Burlingame, CA) and 3,3'-diaminobenzidine (Sigma-Aldrich, St. Louis, MO) were used to visualize antibody binding. The tissues were counterstained with Harris hematoxylin. The Alcian blue/periodic acid-Schiff staining method and hematoxylin-eosin staining were used to detect goblet and Paneth cells, respectively. These results were compared with GATA immunohistochemistry. All the slides were analysed under a light microscope (Leica DMRXA microscope) and reassessed by a pathologist.

### Double immunostaining

The double-staining method was performed on normal paraffin-embedded sections. The sections were prepared as described above, with the following modifications: After staining for GATA-4 (sc-1237) or GATA-6 (sc-9055) with 3,3'-diaminobenzidine, the slides were incubated with the second primary antibody for 1 h at +37°C. The second primary antibody was either a polyclonal goat anti-human chromogranin A antibody (sc-1488, dilution 1:50, Santa Cruz Biotechnology) used to detect neuroendocrine cells in stomach, duodenum, and large intestine, or monoclonal anti-human hydrogen/potassium adenosine triphosphatase (H+/K+-ATPase) antibody against α (119102, dilution 1:50, Calbiochem^®^, EMD Chemicals Inc., Darmstadt, Germany) or β subunit (MA3-923, dilution 1:1000, Affinity BioReagents Inc., Golden, CO) to identify the parietal cells in the stomach. The immunoreactivity was visualised by Vector SG^® ^(Vector Laboratories).

## Results

### GATA-4 and GATA-6 have differential mRNA expression in normal gastrointestinal mucosa

**Figure 1:**
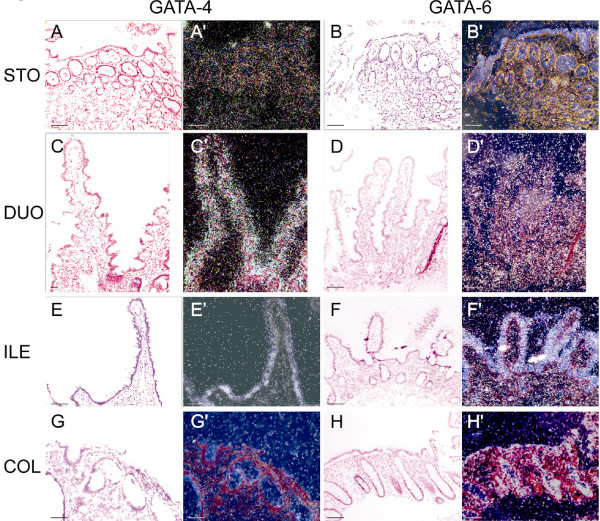
**GATA-4 and GATA-6 mRNA expression in normal gastrointestinal mucosa**. Both bright and dark field *in situ *hybridization views are shown. In the stomach (A, A', B, and B'), GATA-4 mRNA expression is strongest in the middle and superficial layers (A-A'), while GATA-6 mRNA expression is most abundant in the basal two thirds of the mucosa (B-B'). In the duodenum, the enterocytes lining the villi are strongly positive for GATA-4 (C-C'), whereas the GATA-6 signal is more diffuse (D and D'). GATA-4 is no longer detected in the ileum (E and E') and colon (G and G'); the bright lining on the surface of the mucosa is due to tissue autofluorescence (E-E' and G-G'). In contrast, GATA-6 expression is prominent both in the small (D, D', F, and F') and large intestine (H and H') presenting positivity in the surface and crypt enterocytes, and the cells of the lamina propria (D-D', F-F', and H-H'). Scale bar = 50 μm. Abbreviations: STO = stomach, DUO = duodenum, ILE = ileum, COL = colon.

GATA-4 and GATA-6 mRNA expression was first analyzed in the normal gastrointestinal mucosa by *in situ *hybridization. Samples representing stomach, duodenum, terminal ileum, colon, and rectum showed differences in the expression of GATA-4 and GATA-6 along the longitudinal axis and the crypt-villus axis. GATA-4 mRNA was abundant in the superficial two thirds of the mucosa of the proximal gastrointestinal tract (Fig. 1A-A' and 1C-C') but was absent from the distal gut (Fig. 1E-E' and 1G-G'). In contrast, GATA-6 mRNA was highly expressed in the basal regions of the mucosa along the whole gastrointestinal tract, diminishing somewhat towards the distal end (Fig. 1B-B', 1D-D', 1F-F', and 1H-H', data on rectum not shown).

### GATA-4 and GATA-6 protein expression show cell type specificity along the normal human gastrointestinal tract

**Figure 2:**
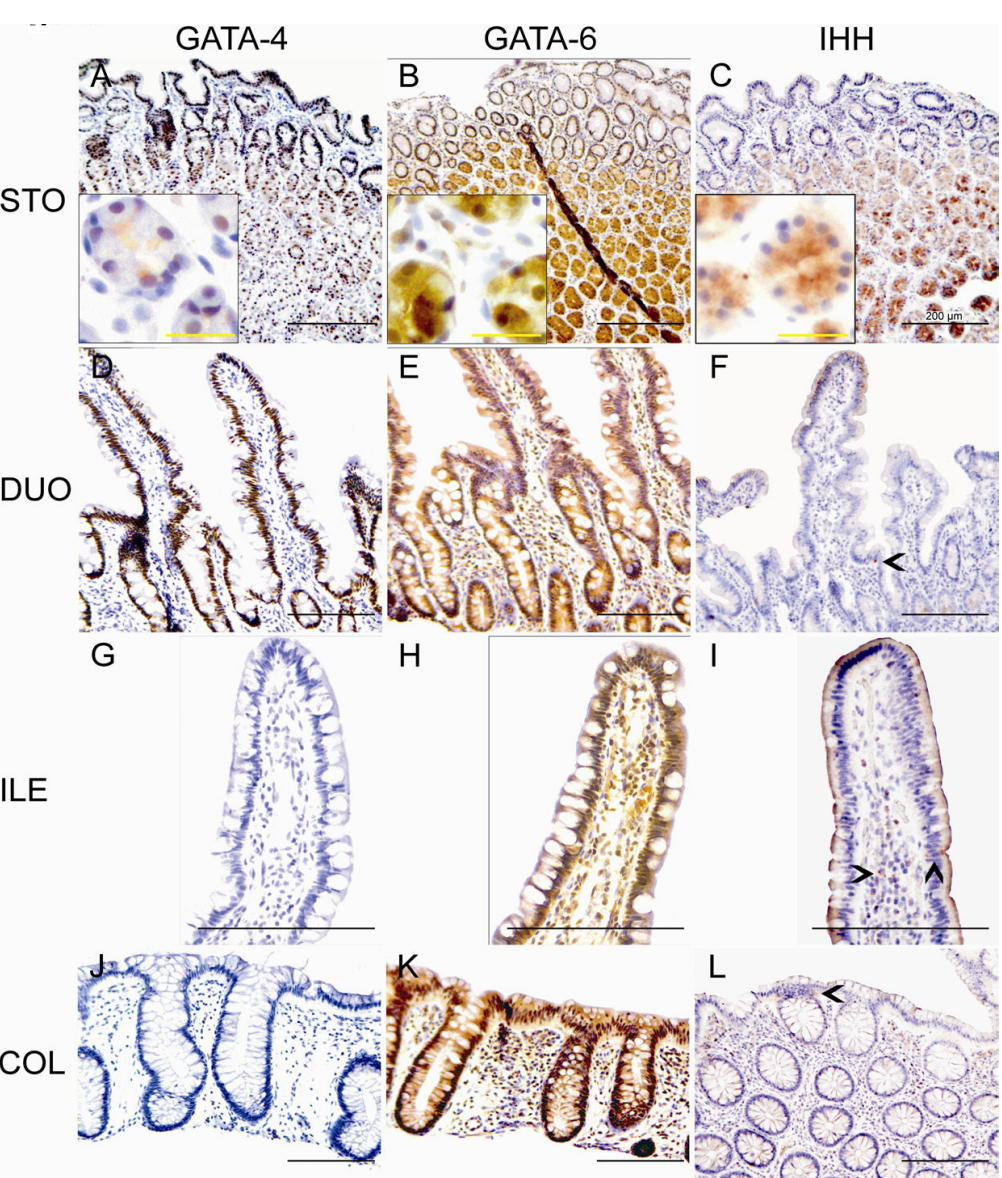
**GATA-4, GATA-6, and Ihh protein expression in normal gastrointestinal mucosa**. Brown nuclear staining indicates positivity for GATA-4 and GATA-6, and brown cytoplasmic color positivity for Ihh. In contrast to GATA-4 (A), GATA-6 (B) and Ihh (C) are strongly expressed at the bottom of the gastric glands (A, B, and C with insets). In the duodenum (D and E), both GATA-4 and GATA-6 are abundant in the villus enterocytes (D and E). In the ileum (G and H) and colon (J and K), GATA-4 is undetectable (G and J), whereas GATA-6 is abundant in the enterocytes, especially in the crypts (H and K). Some GATA-6 positivity is detected also in the lamina propria. Ihh expression is intense in intraepithelial neuroendocrine cells of the small intestine. Positivity can also be seen in some inflammatory cells of lamina propria, though non-specific absorption by plasma cells cannot be ruled out (F and I, arrowheads). In the colon, the enterocytes and the superficial compartments of the lamina propria are weakly positive for Ihh (L, arrowheads). Scale bars: Black = 200 μm and yellow = 25 μm. Abbreviations: STO = stomach, DUO = duodenum, ILE = ileum, COL = colon.

**Figure 3:**
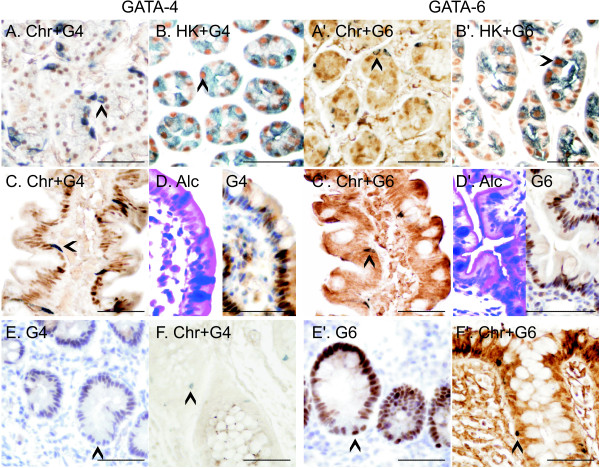
**Identification of different cell types expressing GATA-4 and GATA-6 proteins in normal gastrointestinal mucosa**. Staining for chromogranin A (ChrA) (blue-greyish cytoplasmic staining in A, A', C, C', F, and F') was used to detect neuroendocrine cells and staining for H+/K+-ATPase α (HK) (blue-greyish cytoplasmic staining in B and B' to detect parietal cells. The samples were double-stained for GATA-4 (G4) or GATA-6 (G6) (brown nuclear staining). The GATA proteins are not expressed in the neuroendocrine cells of the stomach, duodenum, or colon (A, A', C, C', F and F' respectively; arrowheads). GATA-4 is detected in the H+/K+-ATPase α presenting parietal cells (B). GATA-6 is positive in approximately two thirds of the parietal cells positive for H+/K+-ATPase α (B', arrowheads). Some duodenal goblet cells, stained deep blue by the Alcian blue method (Alc) (D'), and most Paneth cells are positive for GATA-6 (E', arrowhead) showing no immunoreactivity for GATA-4 (D and E, arrowheads). Scale bar = 50 μm.

Immunostaining was used to delineate the gastrointestinal cell types expressing GATA-4 and GATA-6. GATA-6 immunoreactivity was widespread in the mucosa. In the corpus, GATA-6 expression was abundant in the glands, consisting of chief cells and parietal cells (Fig. 2B with inset). In the antrum and pylorus, the staining for GATA-4 was strongest in the neck and surface epithelium, diminishing towards the base, where GATA-6 exhibited moderate immunoreactivity. In the duodenum, there was no significant GATA-4 immunoreactivity in the Brunner glands, which were positive for GATA-6 (data not shown), but enterocytes lining the villi were highly GATA-4 positive (Fig. 2D). GATA-6 predominated in the crypts of Lieberkühn (Fig. 2E). The mucosa of the terminal ileum (Fig. 2G), colon (Fig. 2J) and rectum (data not shown) generally lacked GATA-4 protein. In 3 out of 17 ileum and colon samples, however, a faint staining for GATA-4 was detected in the epithelium adjacent to a lymph node. Distinct positivity for GATA-6 was detected especially in the bottom of the crypts of the distal gut (Fig. 2K). It is noteworthy that neither GATA-4 nor GATA-6 was found in neuroendocrine cells along the gastrointestinal tract (Fig.3A, 3A', 3C, 3C', 3F, and 3F'). Thus, GATA-4 and GATA-6 presented a cell-specific expression pattern throughout the gastrointestinal tract (Table 3). There were no age-dependent differences in the expression patterns.

**Table 3:** The expression of GATA-4 and GATA-6 in different cell types of the gastrointestinal mucosa on the basis of conventional and double immunohistochemistry

Tissue	Cell type	GATA-4	GATA-6
Stomach			
Corpus			
	Surface epithelial cells	++	++
	Neck cells	+++	++
	Parietal cells	+++	++
	Neuroendocrine cells	-	-
Antrum			
	Surface epithelial cells	++	+
	Neck cells	+++	++
	Glandular cells	++	+++
	Neuroendocrine cells	-	-
Duodenum			
	Villus enterocytes	+++	++
	Crypt enterocytes	++	+++
	Neuroendocrine cells	-	-
	Goblet cells	-	+
	Paneth cells	-	++
Colon			
	Surface colonocytes	-	+
	Crypt cells	-	+++
	Goblet cells	-	++
	Neuroendocrine cells	-	-

The expression patterns based on the number of positive cells detected:

- no expression,

+ low expression; <20% of the cells positive

++ moderate expression; 20–60% of the cells positive

+++ abundant expression; >60% of the cells positive

### Ihh is expressed in normal gastrointestinal mucosa

In the stomach, Ihh expression resembled that of GATA-6; the glandular region, especially in the corpus, showed strong immunoreactivity for Ihh (Fig. 2C). Apart from intense Ihh expression in the neuroendocrine cells throughout the GI tract (Fig. 2F and 2I, arrowheads), the gut mucosa was mostly Ihh-negative. Staining along the brush border of the villus was considered non-specific. Only occasional villus and crypt enterocytes of the small intestine (Fig. 2F) as well as colonocytes and lamina propria of the superficial colon mucosa showed immunoreactivity for Ihh (Fig. 2L, arrowhead).

### GATA-4, GATA-6, and Ihh expression is induced in Barrett's esophagus and intestinal metaplasia of the stomach

**Figure 4:**
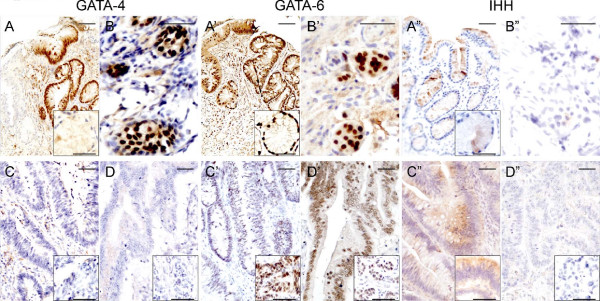
**GATA-4, GATA-6, and Ihh protein expression in neoplastic gastrointestinal mucosa**. GATA-4, GATA-6, and Ihh are strongly expressed in intestinal metaplasia of the stomach (A, A', and A", respectively). The insets in A, A' and A" represent magnifications of cardiac glands. GATA-4 and GATA-6 are strongly expressed in neuroendocrine cell hyperplasia (B and B', respectively) in contrast to no or little expression of Ihh (B"). GATA-4 is detected neither in colon adenomas (C) nor carcinomas (D). The expressions of GATA-6 and Ihh are weak in adenomas, but enhanced in high grade dysplasias (C' and C" with insets). Little GATA-6 is found in the carcinoma tissue (D'). Ihh is not present in colon carcinoma (D"). Scale bar = 50 μm.

GATA-4 and Ihh were not found in normal squamous epithelium of the esophagus, while GATA-6 was expressed in the epithelium near the gastroesophageal junction (data not shown). Barrett's esophagus (data not shown) as well as intestinal metaplasia of the stomach were nevertheless highly positive for GATA-4, GATA-6 and Ihh (Fig. 4A, 4A', and 4A").

### GATA-4 and GATA-6, but not Ihh, are strongly expressed in hyperplastic neuroendocrine cells of atrophic stomach mucosa

Neuroendocrine cell hyperplasia was present in 3 atrophic gastric samples. As described above, normal neuroendocrine cells expressed neither GATA-4 nor GATA-6 but were positive for Ihh. On the other hand, hyperplastic neuroendocrine cells strongly expressed both GATA-4 and GATA-6 (Fig. 4B and 4B'). Interestingly, Ihh expression was weak or absent in these hyperplastic clusters of neuroendocrine cells (Fig. 4B").

### GATA-6 and Ihh are expressed in dysplastic lesions and adenomas of the colon

No expression of GATA-4 was detected in dysplastic lesions or adenomas of the colon (Fig. 4C). In contrast, GATA-6 was positive in colon adenomas and low grade dysplasias; moreover, GATA-6 expression was intense in high grade dysplasias (Fig. 4C' with inset). The expression pattern of Ihh in tubulovillous adenomas and epithelial dysplasias resembled that of GATA-6 (Fig. 4C" with inset). Ihh was not detected in serrated adenoma (data not shown).

### GATA-6 expression is low in colon carcinoma

In colon carcinoma, only low GATA-6 expression was detected (Fig. 4D'), and there was no correlation to histological grade of the tumor (Table 4). GATA-6 expression seemed to be diminished in carcinoma cells as compared to the adjacent normal colon mucosa. On the contrary, our results suggest that GATA-6 expression is stronger in the parts of the carcinoma nearest the surrounding stroma, i.e., in areas representing the invasive edge of the tumor (data not shown). GATA-4 and Ihh were not present in any of the colon carcinoma samples studied (Fig. 4D and 4D").

**Table 4:** The expression patterns for GATA-4, GATA-6 and Ihh in gastrointestinal neoplasias

Tissue	Diagnosis	GATA-4	GATA-6	Ihh
Esophagus (n = 9)				
	Barrett's esophagus	+++	+++	++
Stomach (n = 8)				
	Intestinal metaplasia	+++	+++	++
	Neuroendocrine cell hyperplasia	++	+++	-
Colon (n = 15)				
	Adenoma, low grade dysplasia	-	+	+
	Adenoma, high grade dysplasia	-	++	++
	Adenocarcinoma, low/moderate grade	-	+	-
	Adenocarcinoma, high grade	-	+	-

The expression patterns are based on the number of positive cells detected and the intensity of staining:

- no expression

+ individual positive cells or small areas with low expression

++ patches of moderate expression

+++ large areas of moderate or strong expression

## Discussion

Transcription factors play an important role in cell proliferation and differentiation, and consequently in tissue repair. We studied the expression of two GATA transcription factors in human gastrointestinal tract in detail. As our samples were derived from both children and adults, the wide age variation may have influenced the results. It is noteworthy, however, that in murine gastrointestinal tract, GATA-4 and GATA-6 expression patterns are rather stable from birth to adulthood [30].

In mature gastrointestinal mucosa, new cells are continuously generated from multipotent stem cells. Although the exact location of the stem cells of the gastrointestinal mucosa has not been determined, it has been suggested that in the stomach they lie in the isthmus, whereas in the intestine they may be located deeper in the crypts. The proliferating descendants of the stem cells enter the differentiation pathway and migrate into their specific sites in the mucosa. The cells eventually undergo apoptosis and are shed into the gut lumen [31]. It is of interest that the strongest GATA-6 expression occurs in the basal regions of the gut mucosa including cells with the highest proliferative capacity. Although GATA-6 expression has earlier been thought to decrease during enterocyte differentiation *in vitro *[10], we found GATA-6 in all mucosal layers, including the areas of highly differentiated cells. Ihh has been suggested to be regulated in part by GATA-6 [21], and we find that the expression patterns of these two factors partly overlap in the gut. In contrast to GATA-6, GATA-4 was localized to more differentiated cells (this study) [10]. In the murine small intestine, GATA-4 expression diminishes towards the villus tips the low expression thus associating with areas of apoptosis [15]. In cardiac myocytes, GATA-4 is related to anti-apoptotic factors [32], and its down-regulation is proposed to be essential for apoptosis [33]. We therefore suggest that the absence of GATA-4 from the villus tip enables enterocyte apoptosis and exfoliation of senescent cells.

In chronic gastrointestinal inflammation, such as esophagitis and gastritis, the renewal of normal tissue is disturbed, possibly leading to neoplastic tissue growth [34]. Interestingly, the expression of GATA-4, GATA-6 and Ihh appear to increase in two precancerous lesions such as Barrett's esophagus and intestinal metaplasias of the stomach. GATA-4 and GATA-6 are also expressed in hyperplastic neuroendocrine cells associated with atrophic gastritis. Some researchers have linked GATA-6 to normal murine neuroendocrine cells [35,36], but we detected neither GATA-4 nor GATA-6 in these cells (this study). The appearance of GATA factors in neuroendocrine cell hyperplasia raises the question of whether they contribute to the progression of neuroendocrine neoplasms. Interestingly, GATA factors have been shown to downregulate *HDC *[37] encoding a histamine synthesizing enzyme found in both normal and, in increasing amounts, in neoplastic neuroendocrine tissues [38].

In contrast to metaplasias of the proximal gastrointestinal tract, GATA-4 is not present in colon tumors, whereas GATA-6 and Ihh are moderately expressed in colon adenomas, but to a much lesser extent in carcinomas. Our results suggest that histological tumor grade does not significantly correlate with the level of expression of GATA-6 in cancer cells, although earlier *in vitro *studies have suggested that GATA-6 expression is strongest in the most undifferentiated colon carcinoma cells [10]. In our preliminary analyses, we found that intense GATA-6 immunoreactivity is characteristic of the border regions of malignant tissue and the invasive parts of the carcinoma. This may well be due to stromal signals that induce GATA-6 in the adjacent tumor regions.

The expression patterns of GATA-4 and GATA-6 in the longitudinal and crypt-villus axes are in accordance with the results of earlier studies on murine gastrointestinal tract [15]. In human fetuses, GATA-4 was found in the small intestine [39], and this expression is sustained in mature mucosa as well (this study). Also an earlier study based on RT-PCR analysis demonstrated the absence of GATA-4 from colon, and its presence in stomach [40]. A gene for hydrogen/potassium adenosine triphosphatase (H+/K+ ATPase) in the stomach, responsible for acid production in the parietal cells, is regulated by the gastrointestinal GATA factors [41]. In the parietal cells, GATA-6 positivity varied from one cell to another. This may reflect the fact that the structure and activity of the parietal cells depend on their developmental stage and location in the glands [31]. In our study, Ihh expression was intense in normal gastric glands, whereas Fukaya et al. [24] found Ihh only in gastric pits. The same Ihh antibodies were used in both studies, but the samples in Fukaya's study were neoplastic and their matched tissues. These differences are likely to explain the discrepancies between the two reports.

GATA proteins have been suggested to regulate genes encoding for TFF and mucin proteins [16,17] which protect the mucosa from harmful exogenous agents and are related to abnormal tissue growth and carcinogenesis [42-44]. It is of interest that both GATA-4 and GATA-6 proteins are present in TFF-expressing pit and surface epithelial cells. We detected GATA-6 also in human goblet cells, and others have found GATA-4 in murine goblet cells that express the mucin protein MUC2 [17]. Furthermore, TFF and mucin proteins have been shown to be present in esophageal and gastric metaplasias [45-47], similarly to GATA-4 and GATA-6 (this study). Particularly MUC2 expression in Barrett's metaplasia is considered to indicate a higher risk for carcinoma [46]. Collectively, these data support the interrelationship of GATA proteins, TFF, and mucins in the gastrointestinal endoderm.

A previous study suggested that inactivation of the *Gata4 *gene by methylation could be crucial during carcinogenesis [48]. It is tempting to speculate that GATA-4, in addition to promoting cellular differentiation in the human gastrointestinal tract, is also involved in the suppression of abnormal growth in the proximal gastrointestinal tract. When GATA-4 is inactivated by methylation, the fate of the cells may proceed towards malignant alterations. Likewise, the role of GATA-6 in neoplasias is controversial. In vascular smooth muscle cells, GATA-6 inhibits injury induced hyperplasia [49]. It is noteworthy that GATA-6 is present in adrenocortical adenomas, but diminishes in carcinomas, suggesting that also *Gata6 *may become methylated during tumorigenesis [50]. GATA-6 has been suggested to induce cell cycle arrest [51], to inhibit apoptosis and induce malignant cell growth [52]. Our preliminary results suggest high expression of GATA-6 in the invasive edges of the carcinomas. The enhanced GATA-6 action in these tumor areas may be linked to uncontrolled growth of the tumor cells. More detailed studies are, however, required to establish the role of GATA-6 in the gastrointestinal tumor growth.

## Conclusion

**Figure 5:**
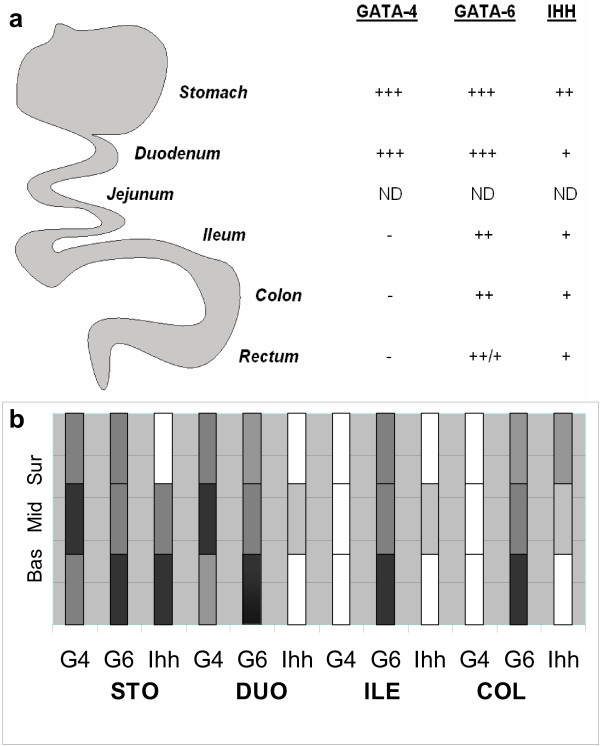
**The expression of GATA-4, GATA-6, and Ihh in gastrointestinal mucosa in a) longitudinal and b) crypt-villus axes**. A summary based on immunohistochemical results: (a) GATA-4 expression diminishes towards the distal end, whereas GATA-6 expression shows only little reduction in the longitudinal axis. Ihh is present very modestly along the gastrointestinal tract, except for the strong expression in the stomach. The expression patterns are based on the number of positive cells detected and the intensity of staining: – no expression, + individual positive cells or small areas with low expression, ++ patches of moderate expression, +++ large areas of moderate or strong expression, ND not detected. (b) GATA-4, GATA-6, and Ihh expression are presented in columns divided into three mucosal layers: surface layer (Sur), middle layer (Mid) and base (Bas). Each section of the GI tract is represented (STO = stomach, DUO = duodenum, ILE = ileum, COL = colon). The intensity of the color demonstrates the intensity of expression along the crypt villus-axis. White depicts undetectable expression.

The specific expression patterns of GATA-4 and GATA-6 and their abundance in the gastrointestinal mucosa (summarized in Figure 5) suggest that they have distinctive and central functions in the gut. Thus, GATA-4 may well be involved in digestive and absorptive functions in the proximal gut, whereas GATA-6 is likely to have a more ubiquitous task throughout the gastrointestinal tract. GATA-4 is expressed abundantly in the cell population programmed to differentiate, and GATA-6, on the contrary, is associated with the proliferative compartment of the mucosal cells. GATA-4 is abundant in intestinal metaplasias and GATA-6 in dysplastic lesions of the colon. Along with GATA-6, Ihh is also induced during abnormal growth. The significance of these factors in gastrointestinal neoplasia remains open, however. The present data offer a firm basis for further investigation of the role of GATA factors and Ihh in normal as well as pathological conditions of human gastrointestinal mucosa.

## Competing interests

The author(s) declare that they have no competing interests.

## Authors' contributions

HH designed the study, performed the experiments, analysed the data and was responsible for writing the paper. MW-O participated in the study design, collection and interpretation of the data, and in writing the paper. KL, MM and ES participated in the collection of the patient samples and data as well as in drafting the manuscript. LCA participated in the study design, collection, analysis and interpretation of the data, and in finalizing the manuscript. MH designed and coordinated the study, interpreted the data, and was responsible for writing the paper. All authors have read and approved the final manuscript.

## Pre-publication history

The pre-publication history for this paper can be accessed here:


